# Pre‐ and postnatal findings with rare congenital anomalies of urinary bladder

**DOI:** 10.1002/ccr3.8590

**Published:** 2024-03-29

**Authors:** Hui Guo, Yanmei Luo, Xinghai Yang, Shigang Cheng

**Affiliations:** ^1^ School of Medicine Wuhan University of Science and Technology Wuhan Hubei China; ^2^ Department of Surgery, Maternal and Child health hospital of Hubei Province Tongji Medical College, Huazhong University of Science and Technology Wuhan Hubei China

**Keywords:** bladder duplication, congenital bladder diverticulum, postnatal, prenatal

## Abstract

Bladder duplication and congenital bladder diverticulum are rare anomalies. We described two boys with rare bladder anomalies found on prenatal ultrasounds. Postnatal investigations and surgical findings confirmed these bladder anomalies. The malformation was associated with other system anomalies. This report of pre‐ and postnatal imaging with surgical correlation contributes to our understanding about these rare bladder anomalies.

## Graphical abstract

The authors report how 2 rare cases of congenital bladder anomalies presented prenatally and postpartum, and after surgical treatment, to help understand this rare bladder anomaly.

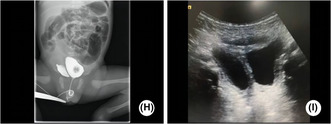

## 1 INTRODUCTION

Bladder duplication and related anomalies are rare congenital abnormalities of the lower urinary tract.^1^ According to the literature, there are less than 80 published cases about bladder duplication.^2^ Bladder duplication can be classified into two categories: incomplete or complete duplication. Incomplete or complete bladder duplication depends on the absence or presence of a duplicated urethra, respectively.^3^, ^4^ Incomplete bladder duplication is defined as an incomplete separation of two urinary bladders by a thick muscular septum that drain into a single urethra. It is often associated with other malformations. Other differential diagnoses include a bladder diverticulum or cyst, a ureteral cyst, and a septated bladder.^5^ Some of the duplicated bladder cases may in fact be examples of other anomalies such as diverticulum, ureterocele, or ectopic ureterocele.^6^, ^7^ A bladder diverticulum is a protrusion of the mucosa through a defect in the destrusor musculature.^8^ Congenital diverticulum is uncommon. Prenatal diagnosis of this anomaly is rare. The purpose of this paper is to record two rare congenital anomalies of urinary bladder and describe the prenatal and postnatal imaging. We hope this might contribute to the literature and assist pediatric urologists to learn more about rare congenital anomalies of the bladder and improve their diagnostic ability.

## 2 CASE REPORTS

### 2.1 Case 1

#### 2.1.1 Case history

**FIGURE 1:**
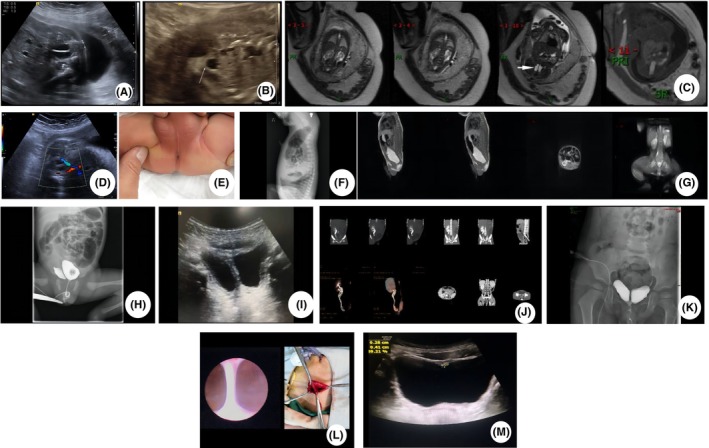
(A, B) Prenatal ultrasound at 27.5 weeks gestation showing abnormal septum in bladder and left multicystic kidney. (C) Prenatal MRI showing duplicated bladder. (D) Repeat prenatal ultrasound at 31 weeks gestation showing abnormal septum in bladder. (E) Physical examination at birth showing anal atresia. (F, G) The inversion radiography and MRI on Day 1 of the life showing anal atresia. (H) Voiding cystourethrography (VCUG) showing a filling defect separating the bladder into two halves. (I) A repeat ultrasound at 1 year old clearly showing abnormal septum. (J) CT urography at 3 years old showing a left renal hypoplasia, a right duplex kidney and incomplete bladder duplication. (K) VCUG at 3 years old showing incomplete bladder duplication. (L) The septum which separate the two bladders was confirmed during the surgery. (M) Ultrasound revealed regular bladder morphology.

This boy was born to a 38‐year‐old G2 mother. The family history was non‐contributory. The mother was admitted to our out‐patient clinic at 27.5 weeks' gestation because of the appearance of left multicystic kidney and abnormal septum in bladder on her routine fetal ultrasound examination (Figure 1A,B). The scan revealed a male fetus with a large bladder. The thickness of the septum was around 2 mm. The other abnormality was polyhydramnios. A fetal MRI confirmed the septum of the bladder which was considered to be a duplicated bladder (Figure 1C). Additionally, there was a left cystic renal dysplasia and a right incomplete duplex kidney. Furthermore, there was an abnormally distended rectum. A follow‐up ultrasound examination at 31 weeks also showed similar findings to the previous one (Figure 1D).

The baby was noted to have an anal atresia at birth (Figure 1E). His physical examination revealed no abdominal mass. On the day of birth, a post‐birth imaging examination was performed to identify whether this boy had associated anomalies. The inversion radiography showed the distance between the distal gas bubbles in the colon and the perineal opening (Figure 1F). MRI imaging also demonstrated the anal atresia, but the MRI didn't show the bladder anomaly because the bladder was empty (Figure 1G). Voiding cystourethrography (VCUG) showed two bladders joined at their low end, and emptied through a common internal urinary urethra (Figure 1H). Anal atresia was corrected surgically on Day 2 of life. The boy had an uneventful recovery after operation. The patient was discharged with a follow‐up to observe for any urinary tract symptoms.

#### 2.1.2 Methods, conclusion and results

A repeat ultrasound was done at 1 year old (Figure 1I). The boy was readmitted with recurrent urinary tract infections at 3 years old. A CT urography examination also showed a left renal dysplasia, a right duplex kidney and incomplete bladder duplication (Figure 1J). He underwent the VCUG again (Figure 1K). The boy performed a left nephroureterctomy and cystoscopy. He was found to have two bladders lying side by side, each receiving a ureter from the ipsilateral kidney. The septum separating the two bladders was excised and the mucosal defect was repaired (Figure 1L). Histopathological assessment revealed a urothelial lining surrounded by smooth muscle which confirmed the diagnosis of incomplete bladder duplication. There were no problems postoperative follow‐up and the boy voided normally. During the first year of follow‐up, post‐surgical ultrasound of the bladder was reviewed again. The scan revealed regular bladder morphology (Figure 1M). There was no hydronephrosis or urinary tract infection for this boy.

### 2.2 Case 2

#### 2.2.1 Case history

**FIGURE 2:**
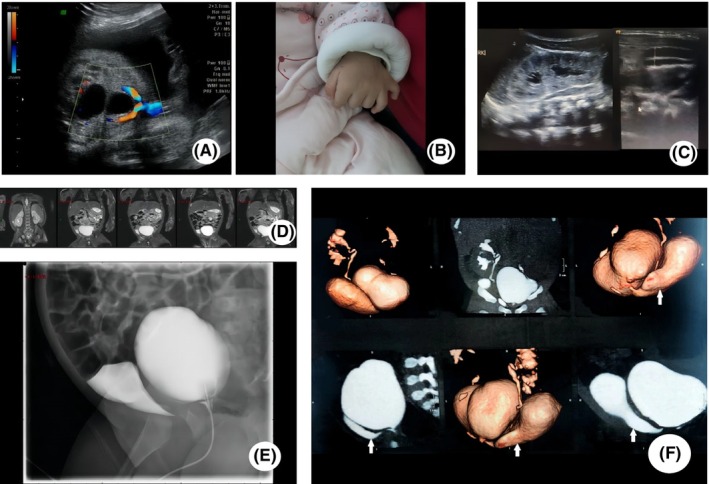
(A) Prenatal ultrasound at 28 weeks gestation showing a cystic structure adjacent to the fetal bladder. (B) Physical examination at birth showing bilateral polydactyly. (C) Repeat prenatal ultrasound showing a right duplex kidney and dilated ureter. (D) MRI at 1 month old showing a cystic mass behind the bladder. (E) Voiding cystourethrography (VCUG) also showing the cystic mass. (F) CT imaging showing the cystic mass and the bladder appeared to unite with a fistulous connection.

A male fetus was diagnosed in ureto by ultrasound at 28 weeks gestation with bladder malformation. The family history was non‐contributory. A cystic structure was visualized adjacent to the fetal bladder (Figure 2A).

#### 2.2.2 Methods, conclusion and results

The boy was noted to have bilateral polydactyly at birth (Figure 2B). A repeat ultrasound showed a right incomplete duplex kidney and dilated ureter (Figure 2C). The MRI was conducted at 1 month of age (Figure 2D). A cystic mass behind the bladder was confirmed. A VCUG also confirmed the mass which was considered to be a duplicated bladder or bladder diverticulum (Figure 2E). We were not sure whether it was bladder duplication or bladder diverticulum. The baby did not undergo any surgeries but had regular checkups at our hospital. At 3 months old, the boy was readmitted with a urinary tract infection. CT imaging showed the cystic mass and the bladder appeared to unite with a fistulous connection (Figure 2F). Because incomplete bladder duplication is an uncommon abnormality, we were confused with the diagnosis. Based on the Abrahamson classification,^1^ after a multi‐disciplinary decision procedure we thought it was a bladder diverticulum. One ureteric orifice would be found in each chamber if it is an incomplete duplicated bladder. He underwent exploratory surgery. During surgery a diverticulum with a narrow‐neck was found and excised with re‐implantation of the right ureter. The right ureter was adjacent to the neck of the diverticulum (Figure 2F). The diverticulum was large and adherent to the surrounding structures. So we chose to re‐implant the right ureter. Histopathological examination displayed urothelial denudation, vascular congestion and chronic inflammatory infiltration represented by lymphocytes. The muscularis propria at the end of the diverticulum attenuated near totally. Seven months later, he was doing well without any new urinary infection events. He was undergoing regular ultrasound checkups in our hospital. The bladder scan showed nothing worth remarking.

## 3 DISCUSSION

As reported by Abrahamson in 1961, bladder duplication and related anomalies are rare anomalies of the lower urinary tract.^1^ Bladder duplication can be classified into complete and incomplete. It was also divided into sagittal (more common) and coronal (less common) duplication. The first case was incomplete duplication in the sagittal plane. Bladder duplications are often associated with other anomalies, which include duplications of the genitalia, such as the vagina and urethra, duplications of the lower gastrointestinal tract, and anorectal ectopia or stenosis.^9^ The boy in our first case also had associated gastrointestinal tract anomalies.

The etiology of bladder duplication is unknown. There are some attempts to explain the embryological development for these anomalies, but a proved description has not yet been determined. A supernumerary cloacal septum that indents the epithelial wall of the bladder has been offered for duplication.^1^ For the cases of congenital bladder diverticulum, the primary cause is thought to be a congenital weakness of the detrusor muscle, most often at the level of the ureterovesical junction.

The prenatal diagnosis of bladder anomalies is a challenge. The variant is commonly associated with other structural duplications.^10^, ^11^ The differential diagnosis of two cystic structures in the lower fetal abdomen should include bladder duplication, bladder diverticulum, ureterocele, hydroureter, ovarian cysts, and a dilated bowel. Both bladder duplication and congenital bladder diverticulum are rare. Sonography can be useful to identify prenatally. There is no diagnostic clinical feature by which these bladder anomalies may be recognized. Some patients present with pyelonephritis, urinary tract infection or bladder outlet obstruction.^12^ We think VCUG can help differentiate among these conditions. We found a few case reports discovered on prenatal imaging. Anomalies of the urinary tract are commonly found in association with congenital anorectal anomalies and should be considered in such cases.

Because of its rarity, both the prenatal follow‐up and the treatment of bladder duplication aren't standardized. According to the literature, various treatment options have been described for incompleted bladder duplication, such as cystoscopic bladder septostomy, neonatal puncture of the bladder septum and complete resection of the septum, or excision of the non‐functional bladder and associated structures.^2^ The more complex anomalies associated with other defects may constitute extremely difficult clinical problems. The aim must be to remove all forms of obstruction in the urinary tract and to eradicate all sources of chronic infection. Optimization of the bladder function and minimization of the risk of infection is the goal of surgical intervention. Bladder duplication does not always require operation if bladder function and drainage are satisfactory.^13^ The prognosis in any particular case will depend on several factors.^1^, ^14^ Besides the bladder anomaly, associated anomalies of the genita‐urinary and other systems will influence the prognosis. Compared to bladder duplication, treatment of congenital bladder diverticulum is based on the symptoms. The management includes observation, endoscopic management, and surgical excision.^15^

## 4 CONCLUSION

We had diagnosed these two bladder anomalies prenatally. If we detected the two cystic structures in the lower fetal abdomen that are adjacent to the umbilical arteries, it should lead us to suspect bladder duplication and other bladder anomalies. We should raise concern for additional gastrointestinal, urogenital, and musculoskeletal anomalies.

## AUTHOR CONTRIBUTIONS

**Hui Guo:** Data curation; investigation; writing – original draft. **Yanmei Luo:** Data curation; investigation. **Xinghai Yang:** Formal analysis; methodology; writing – review and editing. **Shigang Cheng:** Conceptualization; methodology; project administration; supervision; writing – original draft; writing – review and editing.

## FUNDING INFORMATION

This research did not receive any specific grant from funding agencies in the public, commercial, or not‐for‐profit sectors.

## CONFLICT OF INTEREST STATEMENT

The authors declare no competing interests.

## ETHICS STATEMENT

This study was approved by the Human Ethics Committee of Maternal and Child Health Hospital of Hubei Province (2023‐IEC‐088). We certify that the study was performed in accordance with the 1964 Declaration of Helsinki and later amendments.

## CONSENT

Written informed consent was obtained from all the participants prior for the publication of this case report and any accompanying images. The participants had confirmed to the same terms outlined in Wiley's standard consent form. All authors agree to review and publish a version of the case report by Clinical Case Reports.

## Data Availability

All data generated or analyzed during this study are included in this published article and its supplementary information files.
